# Growth Stage of *Alopecurus myosuroides* Huds. Determines the Efficacy of Pinoxaden

**DOI:** 10.3390/plants10040732

**Published:** 2021-04-09

**Authors:** Ana Pintar, Zlatko Svečnjak, Valentina Šoštarčić, Josip Lakić, Klara Barić, Dragojka Brzoja, Maja Šćepanović

**Affiliations:** 1 Department of Weed Science, University of Zagreb Faculty of Agriculture, Svetošimunska Cesta 25, 10 000 Zagreb, Croatia; vsostarcic@agr.hr (V.Š.); jlakic@agr.hr (J.L.); kbaric@agr.hr (K.B.); dbrzoja@agr.hr (D.B.); mscepanovic@agr.hr (M.Š.); 2 Department of Field Crops, Forages and Grasslands, University of Zagreb Faculty of Agriculture, Svetošimunska Cesta 25, 10 000 Zagreb, Croatia; svecnjak@agr.hr

**Keywords:** black–grass, growth stage, pinoxaden dose, delayed application, biomass reduction

## Abstract

*Alopecurus myosuroides* Huds. is an important pinoxaden-resistant grass weed in many countries of Europe. Recently, the low efficacy of pinoxaden was reported in winter cereals in Croatia, but a preliminary dose–response trial showed no herbicide resistance for the investigated weed population. Therefore, a two-year experiment was conducted under greenhouse conditions to determine the efficacy of various pinoxaden doses (20, 40 and 80 g a.i. ha^−1^) on weed visual injuries and biomass reduction after herbicide application at different growth stages. As expected, the maximum weed biomass reduction (97.3%) was achieved by applying the highest dose (80 g a.i. ha^−1^) at the earliest growth stage (ZCK 12–14). A pinoxaden dose of 20 g a.i. ha^−1^ resulted in satisfactory weed biomass reduction (88.9%) only when applied at ZCK 12–14. The recommended dose (40 g a.i. ha^−1^) also provided sufficient weed control up to the growth stage ZCK 21–25. Slightly delayed (ZCK 31–32) application of the recommended dose brought about a low weed biomass reduction (60.1%). Double than the recommended dose also failed to provide satisfactory weed control at the advanced weed growth stages (ZCK 31–32 and ZCK 37–39). Thus, reported low efficacy of pinoxaden is most likely because of delayed herbicide application when *A. myosuroides* is overgrown.

## 1. Introduction

*Alopecurus myosuroides* Huds. (black–grass) is a common annual grass weed in winter cereal crops in north-western Europe [1]. It is widespread in regions with climates influenced by the Atlantic [2], but it is also locally found in other north-western European countries on wet loam and clay soils [3]. Black-grass is a highly competitive species that causes significant yield losses in winter cereals [4]. It has been found that the economic threshold for *A. myosuroides* is 5.5 to 15 plants per m^2^ [5,6,7], and in most cases, more than 10 plants per m^2^ will cause significant yields losses [8]. Additionally, the high reproduction rate [9] and high seed vitality under the high-density conditions require efficient weed control methods [10]. The predicted percentage control of *A. myosuroides* required to maintain a static population in the continuous winter cereals is >90% to achieve its long-term control [11]. The low base temperature (0–1 °C) for germination [1,12], combined with a short primary dormancy, gives this weed species a broad emergence spectrum, which further increases its competitiveness [13]. As a weed species whose life cycle reveals a wide range of adaptation to conventional cropping systems [14], *A. myosuroides* peak emergence occurs in early autumn, coinciding with the sowing time and early development stage of winter cereals. Weed emergence is most commonly observed between October and December (80% of the population), and usually hibernates in two developed leaves or in the tillering phase. Plants appearing in autumn are more advanced compared to those emerging in spring, mainly because *A. myosuroides* can undergo a vernalization period during the cold winter months. Vernalization allows plants to enter the reproductive stage earlier, compared to spring individuals that do not go through the vernalization period and, therefore, enter the reproductive stage later. In addition, spring individuals develop fewer shoots compared to individuals developed in autumn [1]. However, this annual weed may produce seeds in both winter and spring crops under a wide range of growing degree days [10]. Therefore, controlling *A. myosuroides* has always been a major challenge, even though several different herbicide groups are approved for use in winter cereals.

Pinoxaden [15] (Axial^®^ 50 EC, Syngenta Crop Protection AG, Basel, Switzerland) is a selective herbicide for the control of annual grass weeds in winter cereals [16]. It belongs to the phenylpyrazoline chemical class, and its mode of action is based on the inhibition of acetyl-CoA carboxylase (ACCase) [17]. The first symptoms of inhibition appear within the first week, such as initial chlorosis, followed by necrosis and death of rapidly growing meristematic tissue. A time period of two to three weeks is usually required for complete control of susceptible species [16]. The repeated use of ACCase inhibitors since the late 1980s has led to resistance issues in different crops. Resistance to pinoxaden has been identified in more than 15 countries, and the most prominent species is *A. myosuroides*, whose resistant biotypes have so far been identified in Germany, Italy, Poland, Spain and the United Kingdom [18].

In Croatia, *Apera spica-venti* is the most common, and *A. myosuroides* the second most common grass weed in winter cereals [19] as well as the most wide-spread on heavy and clayey soils [20] in the area around Slavonski Brod (45°9′14″ N, 18°1′24″ E). Cereal producers in this area regularly use herbicide pinoxaden to control *A. myosuroides*. The official recommendation in Croatia is that pinoxaden is to be applied between the 3-leaf (ZCK 13) and first node (ZCK 31) stage of the grass weeds with the dose of 40 g a.i. ha^−1^ [15]. However, in recent years, producers of winter cereals have complained about the poor or no efficacy of this herbicide, which was also discussed at the annual Croatian Crop Protection Seminar in 2020 [21].

There is no herbicide monitoring program in Croatia, as is the case in many European countries [18]. Consequently, we conducted a bioassay to evaluate the potential resistance of *A. myosuroides* to pinoxaden [22] and results showed that the investigated population was susceptible to pinoxaden (Figure S1). Therefore, a low efficacy of pinoxaden reported by Croatian farmers is to be attributed to other factors.

The main objective of this research was to determine the efficacy of various pinoxaden doses on visual injuries and biomass reduction of *A. myosuroides* after herbicide application at different growth stages.

## 2. Results

### 2.1. Visual Injuries at 7, 14 and 21 Days after Treatment

**Figure 1:**
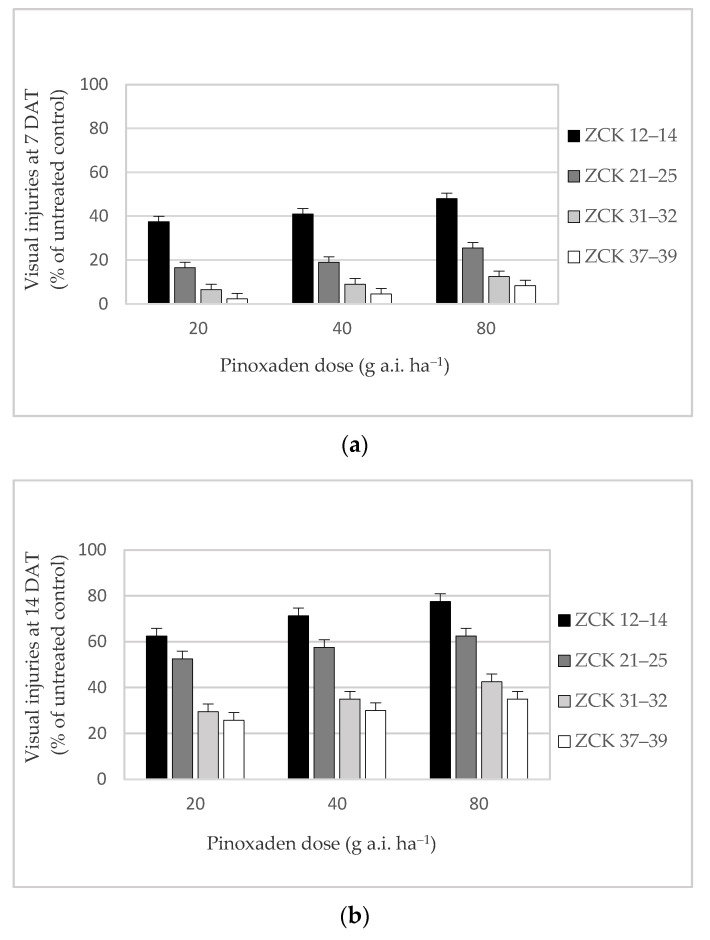

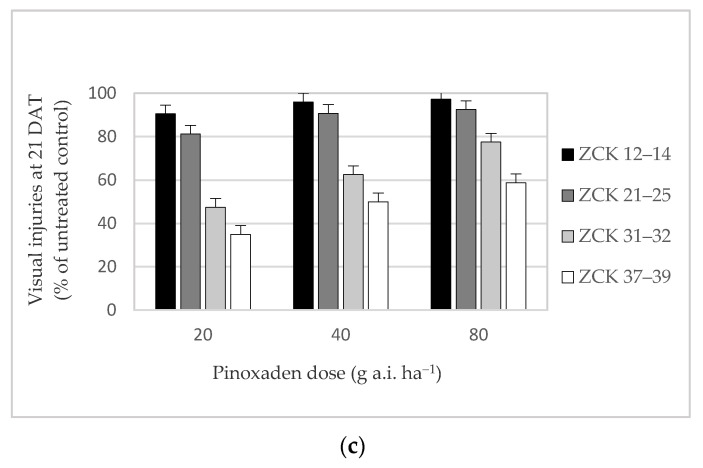
Visual injuries (% of untreated control) of *A. myosuroides* at 7 (**a**), 14 (**b**) and 21 (**c**) days after treatment (DAT) with various pinoxaden doses at different growth stages. Vertical lines indicate a statistical difference according to Fisher’s Least Significant Difference (LSD) test at *p* < 0.05. LSD (**a**) = 3.02; LSD (**b**) = 4.45; LSD (**c**) = 5.14. Greenhouse experiment had four replicates with 10 plants in each pot and was conducted over two years with data pooled across years.

The symptoms of visual injuries were affected by the application doses of pinoxaden and the growth stage of *A. myosuroides*. The progression of injury symptoms was observed with increasing days after treatment (DAT) with pinoxaden for all growth stages of *A. myosuroides*. At 7 DAT, visual injuries were observed on plants treated at ZCK 12–14 and ZCK 21–25, with a maximum of 48.8% and 25.0%, respectively, on plants treated with the double dose (80 g a.i. ha^−1^) of pinoxaden (Figure 1a). Sensitivity of *A. myosuroides* to pinoxaden was significantly reduced when plants were treated at ZCK 31–32 and ZCK 37–39; only about 15% of plants were injured when the double dose of pinoxaden was applied.

At 14 DAT, plant injuries also increased with increasing doses of pinoxaden. These visual injuries were significantly lower on plants treated at more developed growth stages (ZCK 31–32 and ZCK 37–39) compared to the plants treated before (ZCK 12–14) or during (ZCK 21–25) tillering (Figure 1b). Plants treated at ZCK 12–14 showed the highest sensitivity to all applied doses of pinoxaden; injury symptoms ranged from 62.5% after applying pinoxaden at 20 g a.i. ha^−1^ to 77.5% following herbicide application of 80 g a.i. ha^−1^. Plants treated at ZCK 21–25 also showed injury symptoms, with a maximum of 62.5% after the application of double dose of pinoxaden. In contrast, only 42.5% and 35.0% of plants treated at ZCK 31–32 and ZCK 37–39, respectively, were visually injured after application of the double dose of pinoxaden.

The highest visual injuries were determined at 21 DAT (Figure 1c) and were the most visible on plants treated at ZCK 12–14. At this earliest weed growth stage, plants were damaged 90.5, 96.0 and 97.3% after pinoxaden application at 20, 40 and 80 g a.i. ha^−1^, respectively. When pinoxaden was applied at ZCK 21–25, injury symptoms higher than 90% were determined only when the recommended or double dose were applied. When plants were treated at the more advanced growth stages (ZCK 31–32 and ZCK 37–39), visual injuries were less than 80% even when applying the double dose.

### 2.2. Biomass Reduction at 21 Days after Treatment

**Figure 2:**
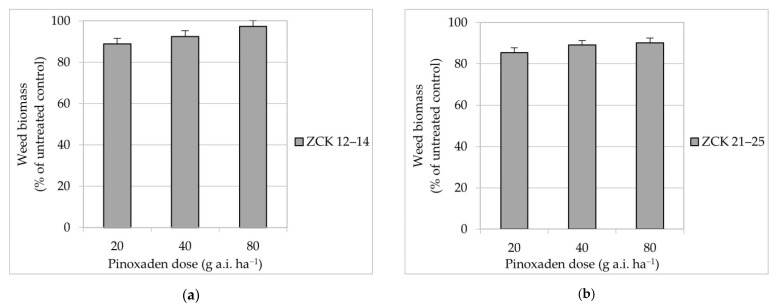

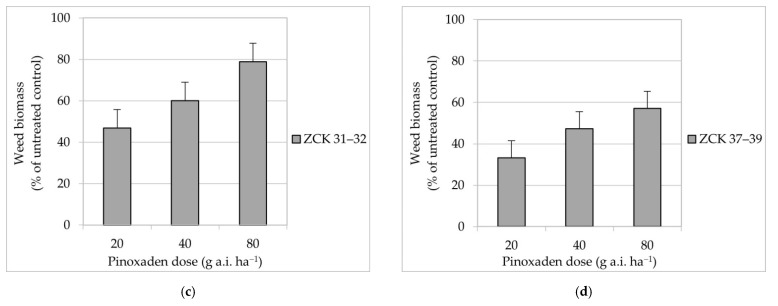
Biomass reduction (% of untreated control) of *A. myosuroides* plants treated with the various pinoxaden doses at ZCK 12–14 (**a**), ZCK 21–25 (**b**), ZCK 31–32 (**c**) and ZCK 37–39 (**d**). Vertical lines indicate a statistical difference according to Fisher’s Least Significant Difference (LSD) test at *p* < 0.05. LSD (**a**) = 2.68; LSD (**b**) = 2.23; LSD (**c**) = 8.93; LSD (**d**) = 8.18.

A significant interaction between herbicide dose and growth stage (data not shown) indicated that weed biomass responses to various pinoxaden doses were influenced by growth stages. Biomass of *A. myosuroides* was greatly reduced following the application of increasing pinoxaden doses at all weed growth stages (Figure 2). Moreover, biomass reduction was lower at advanced weed growth stages. When plants were treated with the reduced (20 g a.i. ha^−1^), recommended (40 g a.i. ha^−1^) or double (80 g a.i. ha^−1^) dose of pinoxaden at ZCK 12–14, the biomass reductions were 88.9, 92.5 and 97.4%, respectively. Applications of recommended and double dose at ZCK 21–25 also reduced weed biomass by approximately 90%. In contrast, when plants were treated at ZCK 31–32 and ZCK 37–39, even the double dose of pinoxaden resulted in a relatively low biomass reduction.

## 3. Discussion

It is well-known that the timing of herbicide application is critical as younger weeds are often more sensitive than those in more advanced stages [23]. This is also demonstrated in our study, which showed a strong potential of pinoxaden to control young *A. myosuroides* plants (before tillering stage). The maximum weed biomass reduction (97.3%) was achieved by applying the double dose (80 g a.i. ha^−1^) at the earliest growth stage (ZCK 12–14). Previous studies also reported differential sensitivity of weeds to herbicides at different growth stages [24,25,26,27]. For example, mesotrion activity was found to be reduced on *Poa annua* L. multitiller plants (five to seven tillers) compared to pretiller plants (three to five leaves) or one-tiller plants [28]. Similarly, *Echinochloa crus-galli* L. P. Beauv. showed the highest sensitivity (89–98%) to all tested herbicides at the four-leaf stage, while application at the six-leaf stage significantly reduced herbicide efficacy (53–64%) [27].

The lowest applied pinoxaden dose (20 g a.i. ha^−1^) in our study brought about a satisfactory control of *A. myosuroides* (Figure 2) only when applied early (ZCK 12–14). However, the recommended dose (40 g a.i. ha^−1^) was required to control weeds sufficiently at full tillering (ZCK 21–25), and this weed growth stage is commonly observed in spring for cereal crops sown in autumn. Despite knowing that pinoxaden is most effective once grass weeds have emerged [29], a spring application of pinoxaden is justified [30] to control plants that have emerged in autumn and spring. Furthermore, unlike other ACCase herbicides in wheat and other cereals, pinoxaden is not antagonized by broadleaf herbicides [31], which makes it attractive for spring application in winter cereals. Previous research showed that *A. myosuroides* is characterized by high seed viability [9] and weed control strategies that prevent flowering and seed production may decrease the soil seed bank contributing to less weed problems in the future.

Our findings clearly demonstrated that pinoxaden is effective for *A. myosuroides* control if applied at the recommended dose up to the full tillering stage. In Great Britain, pinoxaden is recommended to be applied up to the 7 tiller weed growth stage [32]. In contrast, the official recommendation in Croatia is to apply pinoxaden at the dose of 40 g a.i. ha^−1^ between the 3-leaf (ZCK 13) and first node (ZCK 31) stage of the grass weed [15]. Our results showed that the application of recommended pinoxaden dose (40 g a.i. ha^−1^) at slightly delayed weed growth stage (ZCK 31–32) resulted in low weed biomass reduction (60.1%). The morphological and physiological properties of the plant, which determine the absorption, translocation and metabolism of the herbicide, may be a reason for the lower sensitivity of plants at the more advanced growth stages [25,33]. In addition, the process of herbicide degradation is faster in older plants [34]. For example, *Poa annua* and *Poa pratensis* L. multitiller plants metabolized twice as much herbicide mesotrione as pretiller and one-tiller plants [28]. The results of the current study do not allow us to establish a general relationship between leaf structure and herbicide performance, but the lower efficacy of pinoxaden at the advanced growth stage of *A. myosuroides* might be related to epicuticular wax, the content of which increases with the growth stage of the plant [35] and prevents leaf wetting [25].

In Poland, it was found that 60 g a.i. ha^−1^ controlled *A. myosuroides* better than 45 g a.i. ha^−1^ [36]. However, in our research, using the double than recommended dose (80 g a.i. ha^−1^) also failed to provide satisfactory weed control at advanced weed growth stages (ZCK 31–32 and ZCK 37–39). Thus, it appears that the growth stage of *A. myosuroides* determines the efficacy of pinoxaden application. Our findings are consistent with an earlier study in which pinoxaden provided high weed control only when applied no later than the three-tiller stage [29]. Consequently, the low efficacy of pinoxaden against *A. myosuroides* reported recently by Croatian farmers was most likely due to mismanagement issues, i.e., the delayed herbicide application when *A. myosuroides* is overgrown.

In Croatia, pinoxaden is registered for use up to the winter cereal growth stage of ZCK 39 [15]. In applying herbicides, farmers often base their decisions on the development stage of the crop rather than on the weeds [37]. In addition, the most widespread grass weed in winter cereals in Croatia is *A. spica-venti* [19], as also found in Poland [36]. Consequently, decision making process for spring pinoxaden application timing in Croatia is often based on *A. spica-venti* phenology. Previous research [38] indicates that *A. myosuroides* requires the shortest thermal time to reach stem elongation (ZCK 31 and higher) compared to other grass weeds such as *A. spica-venti* and *Lolium multiflorum*. Thermal time from sowing to ZCK 31 averaged 654, 741 and 872 °C for *A. myosuroides*, *L. multiflorum* and *A. spica-venti*. Thus, when *A. spica-venti* is at full tillering, it is expected that *A. myosuroides* is in more advanced growth stages. Croatian farmers typical apply pinoxaden mid-March when average daily temperatures are around 6 °C. Based on the difference in growing degree days requirements, it is evident that *A. myosuroides* may reach ZCK 31 at least 2 weeks earlier than *A. spica-venti*. Therefore, Croatian farmers should avoid focusing on phenology of *A. spica-venti*, which is the most dominant weed in winter cereals. In addition to differences in phenology between *A. spica-venti* and *A. myosuroides*, previous research also found that pinoxaden applied at 45 and 60 g a.i. ha^−1^ controlled *A. myosuroides* slightly weaker compared to *A. spica-venti* [36]. Differences in susceptibility to pinoxaden among other grass weed species was reported in a greenhouse study [39]. Our findings showed that the ability of pinoxaden to control *A. myosuroides* was strongly dependent on whether or not the *A. myosuroides* stem was elongating at the time of application, and consequently, the importance of adjusting the application time based on the growth stage of the weed is highlighted.

## 4. Materials and Methods

### 4.1. Plant Material

The seeds of *A. myosuroides* were collected in July 2019 from the single winter wheat field in Brod-Posavina county, Gornji Andrijevci (45°10′52″ N, 17°52′55″ E), where unsatisfactory control of *A. myosuroides* was reported. The seeds were collected from plants that escaped pinoxaden control, at physiological maturity just before crop harvest. The seeds were cleaned, packed in paper bags and stored in a refrigerator at 4 °C until the beginning of the experiment. In early September 2019, a preliminary trial was conducted to promote germination of *A. myosuroides* by removing chaff and removing chaff +0.2% KNO_3_ to break primary dormancy [40]. Higher germination (90%) was achieved when the chaff from *A. myosuroides* seeds was removed manually.

### 4.2. Experimental Set-Up

The experiment was conducted in the greenhouse at the University of Zagreb Faculty of Agriculture. It was arranged as a randomized complete block design with four replicates and was caried out during two years (2019 and 2020). Seeds of *A. myosuroides* were planted in polystyrene containers (the diameter at the top: 72 mm, the diameter at the bottom: 54 mm, depth: 60 mm) containing humus substrate (Potgraund H, Klasmann, Germany) and placed in a growth chamber (HCP 108, Memmert, Germany) at a constant temperature of 17/11 °C with 70% air moisture and a photoperiod of 14 h of day and 10 h of night [41]. Once the seedlings fully developed true leaf (ZCK 11), 10 seedlings were transplanted in plastic pots (20 × 20 cm) filled with a mixture of humus substrate and sterilized soil. The soil was sampled from the Experimental station of University of Zagreb, Šašinovečki Lug (45°51′00″ N, 16°10′01″ E) and sterilized at 100 °C/30 min to eliminate the germination of all weed seeds [42]. The soil was silty clay loam, with 4.2% humus, 2.9% calcium carbonate and pH 7.7 (H_2_O). The plants grown in pots were regularly watered with an irrigation system (GARDENA MASTER, Australia). The temperatures and photoperiods in the greenhouse were natural, with a minimum temperature of 10 °C during experimentation.

Pinoxaden (AXIAL^®^ 50 EC, Syngenta) was applied with a CO_2_ backpack sprayer, which delivered 200 L ha^−1^ at three different doses: 20, 40 and 80 g a.i. ha^−1^. Pinoxaden applications were performed at four growth stages of *A. myosuroides*: two- to four-leaf stage (ZCK 12–14), one- to five-tiller stage (ZCK 21–25), first to second node stage (ZCK 31–32) and flag-leaf stage (ZCK 37–39). An untreated control for each growth stage was included. *A. myosuroides* plants were visually assessed for the percentage of injuries at 7, 14 and 21 DAT using a scale from 0 (no injury) to 100% (plant death) [43]. Above-ground biomass of *A. myosuroides* was harvested from each pot at 21 DAT, and then dried in an oven (UF 260, Memmert, Germany) at 70 °C for 72 h. At harvest time, untreated plants were at the three- to five-tiller stage (ZCK 23–25), first to third node stage (ZCK 31–33), beginning of heading stage (ZCK 51–53) and full flowering stage (ZCK 65–68) for pinoxaden treatments applied at ZCK 12–14, ZCK 21–25, ZCK 31–32 and ZCK 37–39, respectively.

### 4.3. Statistical Analysis

The statistical analysis was performed with SAS version 8.0 using a Mixed Model Procedure (SAS Institute, Cary, NC, USA). Block and its interaction with the year, growth stage and herbicide dose were random effects. Fixed effects were year, growth stage and herbicide dose. Year effect was nonsignificant so that data were pooled across years. After the significant F-test, the Least Significant Difference (LSD) test for *p* < 0.05 was used to compare the mean values.

## 5. Conclusions

Pinoxaden dose of 20 g a.i. ha^−1^ was effective against *A. myosuroides* only when applied at early growth stage (ZCK 12–14). The recommended (40 g a.i. ha^−1^) dose provided satisfactory weed control up to growth stage ZCK 21–25. The ability of pinoxaden to control *A. myosuroides* decreased rapidly during weed stem elongation despite the use of double than recommended dose. Croatian farmers are advised to adjust the timing of pinoxaden application based on the growth stage of *A. myosuroides*.

## Supplementary Materials

The following are available online at https://www.mdpi.com/article/10.3390/plants10040732/s1, Figure S1: Effect of linearly increasing doses of herbicide pinoxaden on biomass reduction (% of untreated control) of *A. myosuroides* treated at ZCK 13. Vertical line indicates a statistical difference according to Fisher’s Least Significant Difference (LSD) test at *p* < 0.05, LSD = 0.58. Greenhouse experiment had four replicates with 10 plants in each pot.
