# Supplementary figures and images for: Growth Stage of Alopecurus myosuroides Huds. Determines the Efficacy of Pinoxaden
Source: Plants (Basel). 2021 Apr 9;10(4):732. doi: 10.3390/plants10040732 (PMC8068821; doi:10.3390/plants10040732)

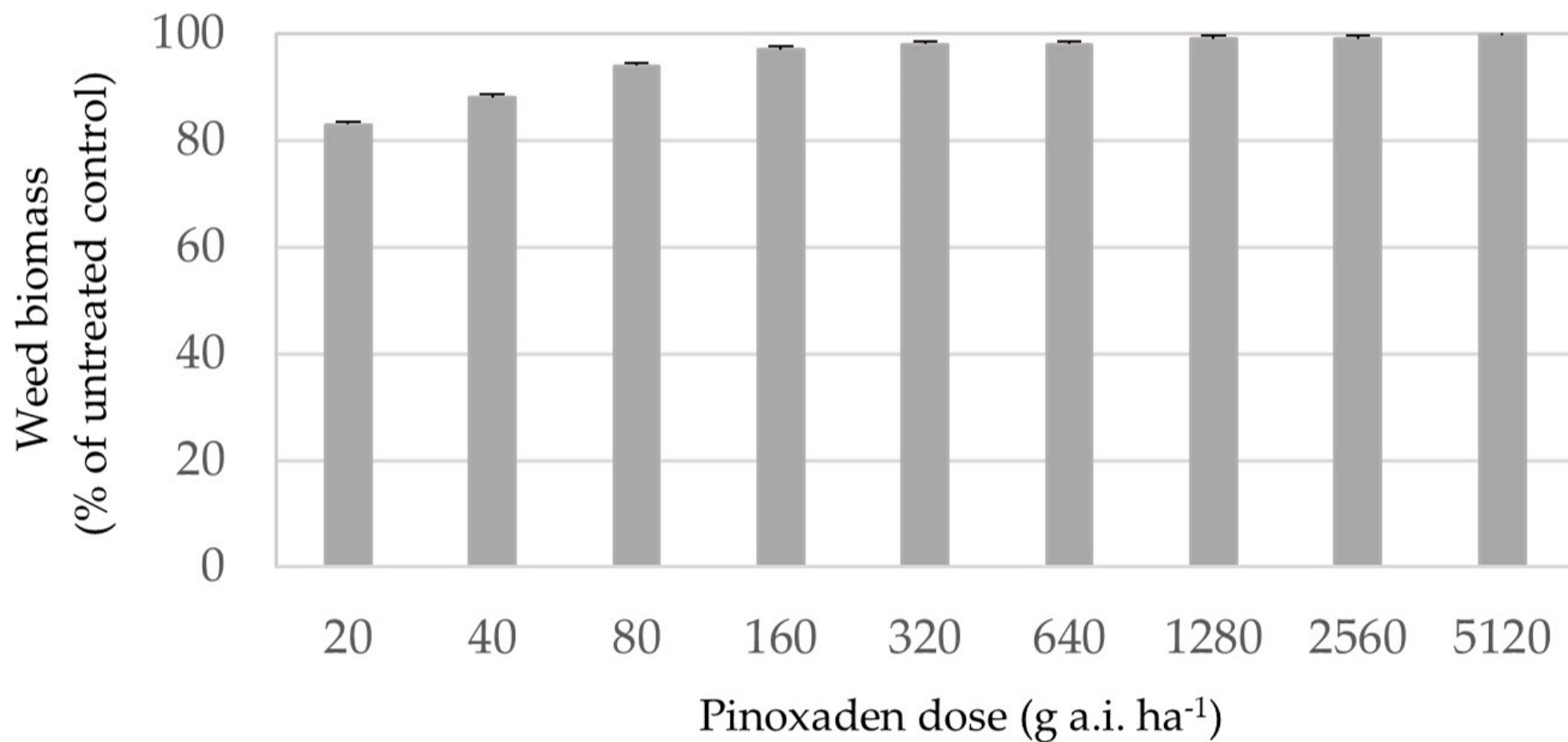

Supplement: Supplementary file 1 [file plants-10-00732-s001.pdf]
